# Feline gastrointestinal eosinophilic sclerosing fibroplasia associated with *Candida albicans*

**DOI:** 10.1002/vms3.70000

**Published:** 2024-08-30

**Authors:** Angel Almendros, Antonio Giuliano, May Tse, Vanessa Rosemary Barrs

**Affiliations:** ^1^ Department of Veterinary Clinical Sciences Jockey Club College of Veterinary Medicine and Life Sciences City University of Hong Kong Hong Kong SAR China; ^2^ Veterinary Medical Centre, City University of Hong Kong Hong Kong SAR China; ^3^ Centre for Animal Health and Welfare City University of Hong Kong Hong Kong SAR China

**Keywords:** candidiasis, eosinophilic, feline gastrointestinal, mast cells, sclerosing fibroplasia

## Abstract

Feline gastrointestinal eosinophilic sclerosing fibroplasia (FGESF) is a rare and poorly understood disease characterised by the infiltration of eosinophils and the development of fibrous tissue within the gastrointestinal tract of cats. A 2‐year‐old female neutered Ragdoll was presented for signs consistent with extrahepatic biliary tract obstruction (EHBO), including jaundice, hyporexia and lethargy. Marked progressive hyperbilirubinemia and mild anaemia were also present. Abdominal ultrasonography suggested a duodenal mass and pancreatitis as the cause of EHBO. Cytopathological results from fine needle aspirates detected mast cells and eosinophils in the duodenal mass and eosinophils in the spleen and the liver, suggestive of a possible mast cell tumour. A cholecystojejunostomy and a duodenotomy were performed to divert the biliary outflow and obtain biopsy samples, respectively. Eosinophilic sclerosing fibroplasia in the duodenal mass and fungal elements in an abdominal lymph node were reported on histopathological examination. A pan‐fungal PCR targeting ITS2 performed on DNA extracted from an abdominal lymph node detected *Candida albicans*. This report adds to the growing body of evidence that FGESF can occur in association with fungal infections.

## Graphical abstract

A Ragdoll cat presented with a duodenal mass diagnosed as FGESF that caused extrahepatic biliary tract obstruction. Fungal elements on a sentinel lymph node were reported as *Candida albicans*.

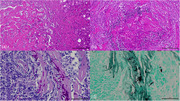

## 1 INTRODUCTION

Feline gastrointestinal eosinophilic sclerosing fibroplasia (FGESF) is a rare disease that causes infiltration of eosinophils and deposition of fibrous tissue to cause mass lesions within the gastrointestinal tract, most commonly involving the stomach and/or small intestine, followed by the ileocaecocolic junction, then the colon (Černá et al., 2024; Craig et al., 2009). Eosinophilic inflammatory changes are associated with the expression of metalloproteinases that lead to matrix remodelling and result in sclerotic changes (Porcellato et al., 2014; Zampieri et al., 2022). Intramural lesions of the gastrointestinal tract are most frequently reported; however, extraintestinal lesions affecting pancreas, mesentery, rectum, lymph nodes or other anatomical sites such as the nasal cavity or intrathoracic structures may be involved (Duclos et al., 2023; Goffart et al., 2022; Kambe et al., 2020; Munday et al., 2014; Thieme et al., 2019; Zampieri et al., 2022). Male cats are more susceptible than females, and Ragdoll cats are overrepresented (Černá et al., 2024; Linton et al., 2015). It has been proposed that bacterial, viral (specifically Feline Herpesvirus 1 – FHV) or fungal infections, foreign bodies or an inherited eosinophilic dysregulation might all play a role in the development of FGESF (Craig et al., 2009; Grau‐Roma et al., 2014; Linton et al., 2015; Thieme et al., 2019).

Although abdominal ultrasonography, computed tomography, endoscopy and exploratory laparotomy are commonly used in the diagnostic investigation of FGESF (Craig et al., 2009; Linton et al., 2015; Weissman et al., 2013), histopathology is necessary for confirmation of eosinophilic infiltration, fibrosis, collagen trabeculae and vascular changes (Craig et al., 2009).

Here we report a rare case of FGESF associated with *Candida albicans* infection in a cat.

## 2 CASE PRESENTATION

A 2‐year‐old female neutered Ragdoll cat was evaluated as a second opinion for inappetence and weight loss of 10‐day duration in association with elevation of liver enzymes and bilirubinuria. The cat had a history of recurrent otitis externa of the left ear and had been treated intermittently with a topical otic preparation containing miconazole nitrate, polymyxin B sulphate and prednisolone acetate (Surolan, Elanco). Additionally, oral oclacitinib (Apoquel, Zoetis) [0.6 mg/kg q 24 h PO] had been prescribed for 3 months prior to presentation for presumptive atopic dermatitis. One month before presentation, the cat had an acute onset of upper respiratory tract signs with serous nasal discharge and sneezing and was treated with oral doxycycline (Vibramycin, Pfizer) [10 mg/kg q 24 h PO] and famciclovir (Famvir, Novartis) [14 mg/kg q 24 h PO] for 10 days.

On physical exam on Day 1 (D1), the cat weighted 4.4 kg with a body condition score of 5/9. She was well hydrated, but her mucous membranes were markedly jaundiced. There was mild discomfort on cranial abdominal palpation, but otherwise, the rest of a physical exam was unremarkable with a heart rate of 170 beats per minute (RR 140–200), a respiratory rate of 26 breaths per minute (RR 15–30) and a temperature of 38.4°C (RR 37.2–39.2). A serum biochemistry panel and a complete blood cell (CBC) count were performed and revealed a marked elevation of liver enzymes and hyperbilirubinemia (Table 1), and a urine analysis revealed bilirubinuria.

**TABLE 1:** Serial haematology and serum chemistry results on a cat with feline gastrointestinal eosinophilic sclerosing fibroplasia (FGESF).

BLOOD test	D1	D15	D21	D28	D35	D42	UNITS	Reference range
HCT/PCV	36.3	29.3	29.5	33.2	32.0	31.0	%	25–48
EOS	**2.74**		**2.57**	**2.92**		0.38	×10^9^/L	0.17–1.57
NEU	7.05		9.12	9.04		7.73	×10^9^/L	2.30–10.29
TP	**73**		**82**	**73**	**85**	**93**	g/L	57–89
GLOB	46	**55**	**56**	47	**52**	**61**	g/L	28–51
ALT	**877**	**480**	**567**	**749**	**3314**	**3259**	U/L	12–130
ALKP	**73**	**112**	**138**	**193**		**189**	U/L	14–111
GGT	**5**	**9**	**13**	**15**	**23**	**19**	U/L	0–4
TBIL	**66**	**88**	**86**	**87**	**139**	**701**	Umol/L	0–15

Abbreviations: ALP, alkaline phosphatase; ALT, alanine aminotransferase; Eos, eosinophils; GGT, gamma‐glutamyl transferase; Glob, globulins; Neu, neutrophils; Tbil, total bilirubin; TP, total protein.

Radiographs of the thorax and abdomen were unremarkable. An abdominal ultrasound revealed enlarged mesenteric lymph nodes, moderate distension of the common bile duct (CBD) and thickening of the duodenal wall at the level of the duodenal papilla, measuring more than 4 mm causing a partial extrahepatic biliary tract obstruction (EHBO); intestinal wall layering was normal, and there were no visible masses or choledocholiths. No abnormal cells or bacteria were detected on the cytopathological exam of bile collected via cholecystocentesis, and bacterial culture of bile was also negative. Pending culture results, oral ursodeoxycholic acid (Ursosan, PRO.MED.CS) [11 mg/kg q 24 h], famotidine (Famotidine, Novitium) [4 mg/kg q 24 h], maropitant (Cerenia, Zoetis) [2 mg/kg q 24 h], mirtazapine (Remeron, MSD) [3.4 mg/kg q 72 h] and metronidazole (Flagyl, Sanofi) [11 mg/kg q 24 h] were prescribed for 2 weeks.

**FIGURE 1:**
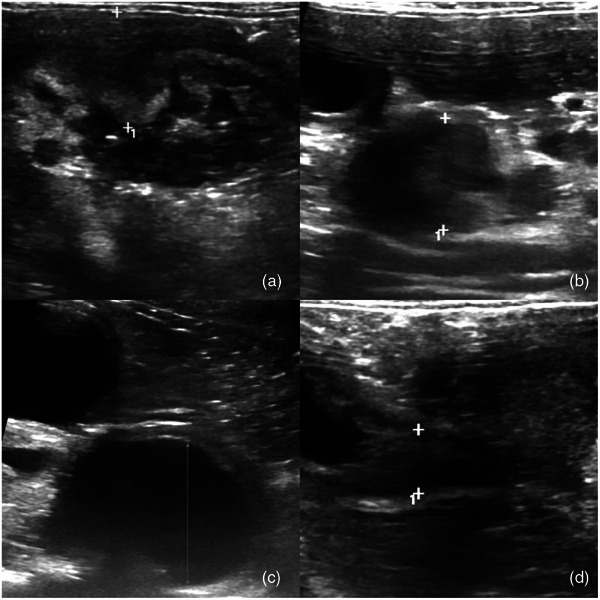
Ultrasonographic images of a cat with feline gastrointestinal eosinophilic sclerosing fibroplasia (FGESF) with a mass effect caused by a marked (9.7 mm) thickening of irregular duodenal wall with loss of normal layering (a), enlarged (14 × 9.3 mm^2^) abdominal lymphadenopathy (b) and marked (13 mm) biliary tract distension (c) caused by partial obstruction of the common bile duct, where abnormal tissue infiltrates the walls (d). The symbol ‘+’ and the ‘arrow’ mark the limits of the structures being measured or mentioned.

At a recheck examination on D15, the cat's appetite had improved, although the body weight was slightly decreased (4.3 kg) and jaundice was more evident. A repeat CBC and serum biochemistry panel revealed hyperglobulinemia, increased liver enzymes, persistent hyperbilirubinemia and eosinophilia (Table 1). Continuation of medical treatment was elected until a revisit on D22 when the client reported that the cat had improved in demeanour and that her appetite and weight had remained stable at 4.25 kg. On physical exam, her mucous membranes were markedly jaundiced, her heart rate was 176 bpm (RR 140–200/min), and her rectal temperature was 38.6°C. Repeat blood tests revealed a markedly regenerative mild anaemia and a severe elevation of total bilirubin as well as other liver enzymes (Table 1). A follow‐up abdominal ultrasound confirmed persistent abdominal lymphadenopathy, further thickening of the duodenal wall that was now 9.7 mm thick and extended into the CBD causing further outflow obstruction and proximal ductal distension (Figure 1).

On D28, the cat was slightly less active but still eating well and had a stable bodyweight of 4.26 kg. A repeat CBC and serum biochemistry panel showed persistent eosinophilia and elevation of liver enzymes and total bilirubin (Table 1). The duodenal mass was more evident on abdominal palpation. A follow‐up abdominal ultrasound examination revealed an unchanged 9.7 mm thick duodenal wall with loss of layering, suggestive of an intramural mass. There was also a more prominent distension of the CBD, persistent cranial abdominal lymphadenopathy and splenomegaly with a mottled appearance. A clotting profile revealed marginally prolonged APTT (140 s; RI <120) and PT (24 s; RI <20). Cytology of a fine needle aspiration (FNA) of the duodenal mass revealed a moderate infiltration of well‐differentiated mast cells, suggesting a possible mast cell tumour, whereas FNA cytology of the spleen revealed eosinophilic infiltration suggestive of eosinophilic splenitis. A hepatic FNA revealed mainly eosinophils with lower proportions of non‐degenerate neutrophils and small lymphocytes. A sample aspirated from the duodenal mass submitted for Feline Coronavirus qRT‐PCR was negative.

An exploratory coeliotomy with tissue biopsies was recommended for confirmation of a potential mast cell tumour, but further investigation was declined by the client. Oral prednisolone (Prednisolone, Sandoz) [2 mg/kg q24h PO] and toceranib (Palladia, Zoetis) [2.3 mg/kg q 56 h PO] were added following an oncological consultation. Vitamin K (Konakion, Roche) [1.5 mg/kg q 12 h PO] was also prescribed for 2 weeks. A revisit on D35 revealed a further increase of total bilirubin and liver enzymes (Table 1). The weight was slightly decreased (4.15 kg), and the appetite remained unchanged. The cat was still bright and alert in the consult room, and otherwise the physical exam was unremarkable.

On D42, the cat's weight had decreased to 4.06 kg, and she was less active and markedly icteric. Her total bilirubin and liver enzymes had increased further (Table 1). An exploratory laparotomy was performed. The intestinal mass was unresectable, and biopsy samples of the duodenum were obtained through a duodenotomy, which was performed to provide access for stenting of the duodenal papilla. However, severe tissue thickening around the duodenal papilla precluded this procedure. A cholecystojejunostomy was performed as a salvage procedure to redirect the biliary outflow.

Unfortunately, the cat developed septic shock and died in the perioperative period, 36 h post‐surgery, following a suspected bacterial leakage from the surgical site. A post‐mortem examination was declined.

**FIGURE 2:**
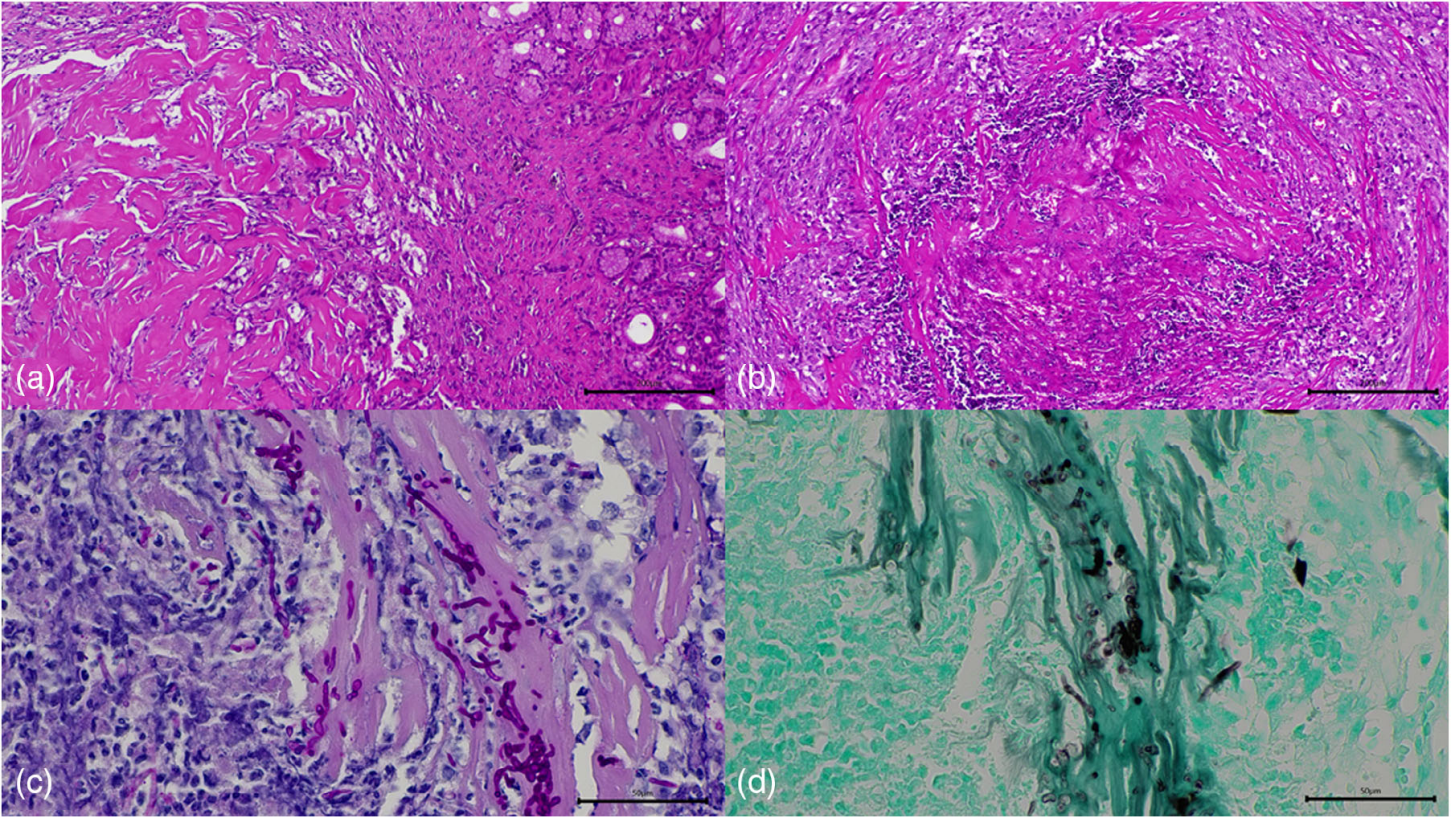
Histopathology images of the fibroplasia replacing the duodenal muscularis (a) and the pancreatic lymph node (b). Haematoxylin and eosin (H&E) 100×. Fungal elements are embedded in the dense fibrous tissue admixed with inflammatory cells in the lymph node. Periodic acid Schiff (PAS) (C) and Grocott methenamine silver (GMS) (D) 400×.

Histopathology of the duodenal biopsies collected at laparotomy revealed the presence of a multifocal, chronic, mastocytic and neutrophilic enteritis with fibroplasia, ulceration, granulation tissue and mineral deposition, consistent with a diagnosis of eosinophilic sclerosing fibroplasia (Figure 2). A biopsy from an abdominal lymph node was described as moderate, chronic, multifocal, eosinophilic, histiocytic and mastocytic lymphadenitis with fibroplasia. Intralesional fungal elements were detected in the lymph node on periodic acid Schiff (PAS) and Grocott methenamine silver (GMS) stains (Figure 2). A pan‐fungal PCR targeting the internal transcribed spacer‐2 region of the region rRNA gene cluster detected the pathogen to be *C. albicans*.

## 3 DISCUSSION

This case report describes a cat with a duodenal mass and enlarged abdominal lymph nodes with histopathological features of FGESF associated with *C. albicans* infection.

Initial investigation included cytopathology, which revealed the presence of mast cells and eosinophils in the intestinal mass, liver and spleen. These findings were suggestive of a possible mast cell neoplasm and led to the initial treatment with corticosteroids and toceranib, which, after further investigation, was initially declined. A potential ‘dry’ or non‐effusive form of feline infectious peritonitis (FIP) was also initially considered, but Feline Coronavirus RNA was not detected in the specimen collected via FNA from the intestinal mass.

The diagnosis of FGESF from the histological examination of tissue biopsies can be difficult. As in this case, misdiagnosis of mast cell neoplasia due to mast cell infiltration and lymphadenopathy has been reported previously in cats with FGESF (Craig et al., 2009; Davidson et al., 2021; Linton et al., 2015; Weissman et al., 2013). Sclerosing mast cell neoplasia was considered an entity previously, but the widely dispersed or perivascular distribution of mast cells makes it inconsistent with neoplasia (Craig et al., 2009). As well as mast cell neoplasia, FGESF has been misdiagnosed as other neoplasms, including fibrosarcoma, lymphoma and adenocarcinoma (Gamble, 2010; Linton et al., 2015; Munday et al., 2014; Schulman & Lipscomb).

Unfortunately, the delay in the diagnosis precluded earlier and more specific treatment to target both the FGESF and the *C. albicans* infection. Prednisolone treatment in this case (1.5 mg/kg/day) was not effective, and treatment was complicated by the concomitant administration of toceranib. In a retrospective multicentre study of 60 cats with FGESF, 59/60 were prescribed prednisolone at a median dose of 1.5 mg/kg/day with 53 (88%) responding to therapy (Černá et al., 2024). Other cases affecting the duodenum, lymph nodes, mesentery and retroperitoneum have also been reported to respond to oral corticosteroids or to combined therapy with ciclosporin (Agulla et al., 2021; Kambe et al., 2020; Thieme et al., 2019). A multimodal approach, including prednisolone in combination with ciclosporin, started earlier might have been more effective to resolve the fibrotic and sclerosing inflammatory response in the present case. However, the combination of prednisolone and cyclosporin is potently immunosuppressive and might have enabled haematogenous dissemination of the *C. albicans* infection. Immunosuppressed human patients are a high‐risk group for *C. albicans* bloodstream infections (Sari et al., 2024).

Similar to other reports (Craig et al., 2009; Linton et al., 2015), the anatomical location in our case might have had a negative impact on prognosis as it caused progressive EHBO that required surgical intervention, but ultimately resulted in sepsis. Additionally, the definitive diagnosis was only possible after surgical biopsies were performed. Surgical excision of the mass would have improved the likelihood of cure; however, as in many other reported cases, the mass was unresectable. This highlights the importance of prompt histopathological confirmation of FGESF.

In the present report, *C. albicans* was identified by PCR, which has been reported in association with FGESF in only one other case, where *C. albicans* was isolated in culture together with *Escherichia coli* and an *Enterococcus* species (Černá et al., 2024). In that case, fungal elements were also detected on PAS stains on histological examination. However, the source of the tissue (gastrointestinal, lymph node or other) was not reported.

Two other cases of FGESF had been described previously in association with fungal infection (Grau‐Roma et al., 2014; Martineau et al., 2023). Mucormycosis was suspected in one case based on the morphology and staining features of fungal elements on haematoxylin and eosin and special stains (PAS and GMS) (Grau‐Roma et al., 2014). Like in our case, no fungal culture was performed due to suspicion of neoplasia at the time of biopsy sampling. In the other report, fungal culture of a proximal duodenal lesion revealed *Rhizopus microsporus*, a siphomycetous fungus also from the *Mucorales* order (Martineau et al., 2023).

Candidiasis is the most common nosocomial mycotic infection in humans (Suleyman & Alangaden, 2021). It has also been reported to cause mass‐like lesions in the pyloroduodenal region with EHBO in people (Alakkam et al., 2019). In cats, *C. albicans* was reported to cause an obstructive intramural duodenal mass in a single case report (Duchaussoy et al., 2015; Grooters, 2003; Souto et al., 2020). In the present case, *C. albicans* was only detected in abdominal lymph node tissue but not in the duodenal biopsy. Despite this, the origin of *C. albicans* was likely gastrointestinal. The cat had been treated for chronic dermatitis and otitis externa for over 3 months with oclacitinib, a drug that modulates the activity of Janus kinase 1 and has immunosuppressive properties (Moore et al., 2022). Oclacitinib was associated with a fatal case of toxoplasmosis in an immunosuppressed cat after 5 months of treatment (Moore et al., 2022). Janus kinase inhibitors modulate cytokine expression and immune responses and have been reported to increase the risk of infections in humans (Winthrop et al., 2016) and to reduce survival in mice infected with *C. albicans* (Chen et al., 2017). Whether the prolonged use of oclacitinib or the use of antibiotic therapy might have facilitated the growth of the fungal pathogen in this case is not known. The pathological role of *C. albicans* in our case, if any, is nevertheless uncertain.

Secondary bacterial, protozoal or fungal infection may occur as a consequence of prior damage to small intestinal mucosa (Linton et al., 2015). Conversely, fungi, parasites, certain viruses (FHV) and food allergens have been postulated to be an initial trigger for eosinophilic inflammation associated with the release of mediators that lead to fibrosis (Cox et al., 1995; Craig et al., 2009; Gleich, 2000). *Mucorales*, opportunistic microorganisms that inhabit soil, water and putrefying organic material, were detected in two cases of FGESF; their presence was speculated to have been caused by ingestion, with colonisation and invasion occurring secondary to pre‐existing gastrointestinal barrier disruption (Grau‐Roma et al., 2014; Martineau et al., 2023). Whether these are primary or secondary infections, it is plausible that these microorganisms play a role in the development of FGESF, as has been postulated previously (Craig et al., 2009).

The presence of *C. albicans*, in this case, was only confirmed after surgical biopsies were taken. It is possible that FNA cytology of the enlarged lymph node might have demonstrated fungal infection earlier in the diagnostic investigation, which might have led to the addition of antifungal, yet it is unknown whether this would have changed the outcome.

## 4 CONCLUSION

This case adds to the growing body of evidence that fungal pathogens can be detected in FGESF. In this study, we highlight the importance of prompt diagnosis and timely treatment in order to increase the success outcome when treating this challenging disease. Further research to better understand the role of microorganisms in this enigmatic condition is needed.

## AUTHOR CONTRIBUTIONS

**Angel Almendros**: Conceptualisation; methodology; formal analysis; writing–original draft. **Antonio Giuliano**: Methodology; formal analysis; data curation; writing–review and editing. **May Tse**: Data curation; writing–review and editing. **Vanessa Rosemary Barrs**: Methodology; formal analysis; writing–review and editing.

## CONFLICT OF INTEREST STATEMENT

The authors declare no conflicts of interest.

### ETHICS STATEMENT

The authors confirm that the ethical policies of the journal, as noted on the journal's author guidelines page, have been adhered to. No ethical approval was required as this is a retrospective study with no original research data but analysing only history records.

### PEER REVIEW

The peer review history for this article is available at https://publons.com/publon/10.1002/vms3.70000.

## Data Availability

The data that support the findings of this study are available on request from the corresponding author. The data are not publicly available due to privacy or ethical restrictions.
